# Acceptance and hesitancy to receive COVID-19 vaccine among university students in Egypt: a nationwide survey

**DOI:** 10.1186/s41182-023-00509-9

**Published:** 2023-03-09

**Authors:** Samar Tharwat, Ahmed Mohammed Saad, Mohammed Kamal Nassar, Dalia Kamal Nassar

**Affiliations:** 1 Rheumatology & Immunology Unit, Department of Internal Medicine, Faculty of Medicine, Mansoura University, El Gomhouria St, Mansoura, Dakahlia Governorate Egypt; 2 Faculty of Medicine, Mansoura University, Mansoura, Egypt; 3 Mansoura Nephrology & Dialysis Unit (MNDU), Department of Internal Medicine, Faculty of Medicine, Mansoura University, Mansoura, Egypt; 4 Medical Microbiology and Immunology Department, Faculty of Medicine, Mansoura University, Mansoura, Egypt

**Keywords:** COVID-19, Vaccine acceptance, Vaccine hesitancy, University students, Barriers, Knowledge

## Abstract

**Background:**

The public's willingness to be vaccinated will determine the success of the COVID-19 vaccination program. The aim of this study was to identify acceptance and hesitancy to receive COVID-19 vaccine among university students in Egypt, assess their level of knowledge about COVID-19 vaccine and identify factors that influence their intention towards COVID-19 vaccination.

**Methods:**

A standardized and self-administered questionnaire was distributed among university students allover Egypt. The questionnaire included sociodemographic data, intention to receive COVID-19 vaccine, knowledge and beliefs about it and status of COVID-19 vaccination. Logistic regression analysis was used to determine factors associated with COVID-19 vaccine acceptancy.

**Results:**

A total of 1071 of university students were involved, with a mean age of 20.51 years (SD = 1.66) and 68.2% were females. COVID-19 vaccination acceptability rate was 69.0% while hesitancy was 20.8% and resistancy was 10.2%. Median knowledge score of 4 out of 8 (IQR = 8). The main motivation for vaccine acceptance was fear of being infected (53.6%) and desire to get back to normal life (51.0%) while the main barriers against getting vaccinated were being afraid of serious side effects. Univariate regression analysis revealed an increasing likelihood of vaccine acceptancy associated with an active lifestyle (OR 1.35, 95% CI 1.04–1.75, *p* = 0.025), a high knowledge score (OR 1.53, 95% CI 1.42–1.66, *p* < 0.001), and positive vaccine beliefs.

**Conclusion:**

There is a high rate of acceptability of COVID-19 vaccination among university students. Vaccine acceptability is associated with an active lifestyle, a high knowledge score and positive vaccine beliefs. Educational campaigns and efforts aiming to raise awareness about safety and efficacy of COVID-19 vaccines need to be directed to this important population.

## Background

On March 11th, 2020, World Health Organization (WHO) declared Coronavirus disease 2019 (COVID-19) as a global pandemic [1]. As of 16th of September, 2021, WHO reported diagnosis of 226 million cases of COVID-19 and death of approximately 4.5 million people [2]. Since its discovery, unprecedented efforts to develop COVID-19 vaccines [3].

Till 19th of August, 2021, there had been twenty-four candidate vaccines submitted to WHO to be validated for emergency use, of which 7 have been approved [4]. The Egyptian drug authority has given emergency use authorization for six vaccines in Egypt: four non-replicating viral vector vaccines (Gamaleya Sputnik V, Janssen (Johnson & Johnson), Oxford/AstraZeneca AZD1222, Covishield (Oxford/AstraZeneca) and two inactivated vaccines (CoronaVac from Sinovac and BBIBP-CorV from Sinopharm) [5].

Key elements for protection against COVID-19 infection could be established by following precautionary measures to avoid infection along with mass vaccination [6]. However, low confidence levels in vaccination and its effectiveness is concerning [7].

In 2019, WHO designated ten threats to global health, which included a growing and widespread phenomenon called ‘‘vaccine hesitancy” [8]. Vaccine hesitancy refers to a ‘‘delay in acceptance or refusal of vaccines despite the availability of vaccination services” [9]. It represents a threat to public health allowing for the emergence of subpopulations of unvaccinated patients and occurrence of outbreaks of vaccine-preventable diseases [10].

As for COVID-19 vaccines, there is an urgency for mass vaccination in the light of increasing numbers of cases and emerging variant strain of the virus [3]. University students are considered as a perceptive group of young adults and their intention to receive COVID-19 vaccines should be assessed. This subpopulation are presumably knowledgeable with an open minded attitude and faster response to public health issues with increased risk of infection due to presence in crowded settings [11, 12]. Moreover, they can have a leading role, especially medical students, in addressing vaccine hesitancy through promoting clear scientific messages and positive attitudes towards vaccination [13].

Nevertheless, information about university students’ acceptability, beliefs and knowledge of COVID-19 vaccination is lacking [14]. Knowing that would be helpful to estimate vaccine uptake and allow for development of strategies in order to improve acceptability and overcome hesitancy problem [6]. To our knowledge, vaccine acceptability among university students together with actual translation into vaccine uptake has not been assessed in Egypt.

This study aimed at estimating the intention to receive COVID-19 vaccine among university students in Egypt and their knowledge, beliefs, and attitude towards COVID-19 vaccination together with the actual uptake of the vaccine.

## Methods

### Study design and setting

The study was a cross-sectional survey study by completing self-administered online questionnaire created on Google forms. All students in different Egyptian universities aged more than 18 years were eligible for participation in the study. The students were identified through their social pages and official groups on social networking sites such as Facebook and WhatsApp. The questionnaire had been randomly delivered to all potential participants using social media platforms for about 1 month starting from 20th of July till the 20th of August 2021. Then, they were directed to a website that briefly described the aim of the research and instructions to complete the questionnaire. Participants were assured of anonymity and confidentiality of data. Informed consent: includes the fact that participation is voluntary, anonymous, and confidential, instructions for filling out the questionnaire, an invitation to join, and an informed consent option. Only those who choose "accept to participate" will be able to complete the rest of the questionnaire. The link led to Google Forms for anyone who accepted participation in the study. Answering all the questions and submitting them was also considered as a consent to participating in the study.

### Calculation of sample size

The online sample size calculator RaoSoft® was used to calculate the appropriate sample size. Based on an estimated population of 3 million students in Egyptian universities [15], 50% predicted response, 5% margin of error and 95% confidence level, the minimum sample size was 385 participants.

### Questionnaire structure

The questionnaire was developed by the researchers according to extensive review of literature [16–18]. Multiple-choice questions were included in the questionnaire which was initially structured in English then translated into Arabic. Pilot study was performed on 12 students to test the questionnaire and they were subsequently excluded from the data analysis.

The questionnaire consisted of questions collecting information about: sociodemographic data, smoking habits, health status and history of previous COVID-19 infection, hospitalization, or ICU admission either personal or of someone in their social circle. In addition to asking about self-rated knowledge and beliefs about COVID-19 vaccine, eight questions were asked to assess their knowledge about COVID-19 vaccine. Each correct answer was given one mark and a total score out of 8 was calculated for to assess knowledge in all students. Knowledge score 4 out of 8 demonstrated inadequate knowledge [16]. Also, participants were questioned about their intention to get COVID-19 vaccination, reasons for or against being vaccinated.

Lastly, the respondents were questioned about their COVID-19 vaccination status. For those who had been vaccinated, they were asked about the type of the vaccine received and side effects they experienced, namely, anaphylaxis, skin rash, muscle or joint pain, fever or chills, fatigue or sleepiness, chest pain or palpitations, headache, nausea, vomiting and poor appetite.

**Fig. 1:**
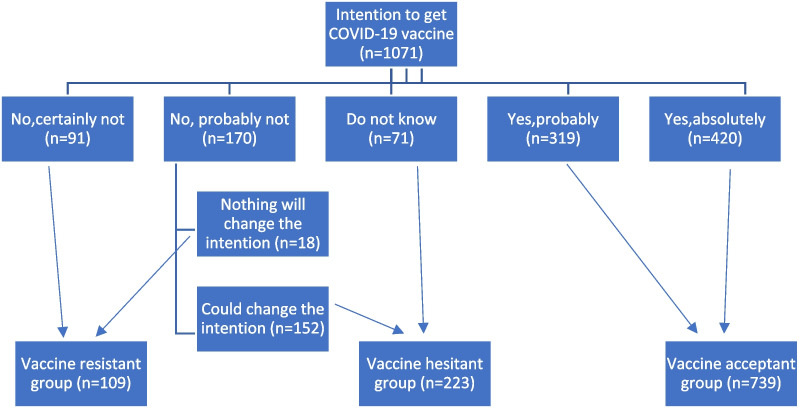
Flowchart of the study groups according to COVID-19 vaccination intention

The participants were classified as the following: vaccine acceptant (VA) if they chose “Yes, absolutely” or “Yes, probably”. If they chose the options “No, probably not” or “I don’t know”, they were considered vaccine hesitant (VH). Vaccine resistant (VR) participants were those who responded as “No, certainly not” or “No, probably not” and “Nothing will change the intention” [18] as illustrated in Fig. 1.

The VA participants were asked about the motivators for COVID-19 vaccination, whereas the VH and VR participants were asked about the impediments to it. Those who identified as VH were questioned about the possibility of altering their intention and the underlying reasons.

### Statistical analysis

All data were analyzed by SPSS (version 21). We assessed participants’ vaccine acceptability as a primary endpoint by five-level Likert scale question asking about their intention to get a COVID-19 vaccine.

Data normality was tested using the Kolmogorov–Smirnov test. Categorical variables were compared using Chi-square or Fisher’s exact test. Continuous variables were expressed as mean and standard deviation (SD) and compared using ANOVA test. Nonparametric data were described as median, minimum, maximum and IQR and were analyzed by Kruskal–Wallis test. Considering that VA is the outcome variable, VH and VR were combined together and logistic regression analysis was used to determine factors related to COVID-19 vaccine acceptancy. The factors were chosen based on variables that demonstrated a significant association with acceptancy, such as college, an active lifestyle, knowledge score, and attitude toward COVID-19 vaccination.

## Results

The study initially included 1130 university students who responded to the questionnaire and after exclusion of incomplete or invalid data, 1071 university students were recruited from 28 different universities allover Egypt constituted the study participants. The mean age of 20.51 ± 1.66 years, 730 (68.2%) were females and 341 (31.8%) males. Other sociodemographic data are shown in Table 1.

**Table 1:** Sociodemographic data of study participants according to their intention to receive COVID-19 vaccination (*n* = 1071)

Variables Mean ± SD or n (%)	Total (*n* = 1071)	VA group (*n* = 739)	VH group (*n* = 223)	VR group (*n* = 109)	*p*
Age (years)	20.51 ± 1.66	20.52 ± 1.78	20.51 ± 1.30	20.44 ± 1.56	0.891
Sex					
Females	730 (68.2)	495 (67)	164 (73.5)	71 (65.1)	0.142
Males	341 (31.8)	244 (33)	59 (26.5)	38 (34.9)	
Marital status					
Single	1055 (98.5)	728 (98.5)	220 (98.7)	107 (98.2)	0.942
Married	16 (1.5)	11 (1.5)	3 (1.3)	2 (1.8)	
University					
Upper Egypt	97 (9.1)	62 (8.4)	19 (8.5)	16 (14.7)	0.098
Lower Egypt	974 (90.9)	677 (91.6)	204 (91.5)	93 (85.3)	
College					
Non-medical	171 (16)	106 (14.3)	34 (15.2)	31 (28.4)	0.001*
Medical	900 (84)	633 (85.7)	189 (84.8)	78 (71.6)	
Academic year					
First	182 (17.0)	115 (15.6)	38 (17.0)	29 (26.6)	0.21
Second and third	574 (53.6)	408 (55.2)	116 (52.0)	50 (45.9)	
Fourth and more	315 (29.4)	216 (29.2)	69 (30.9)	30 (27.5)	
Residence					
Rural	528 (49.3)	362 (49.0)	114 (51.1)	52 (47.7)	0.804
Urban	543 (50.7)	377 (51.0)	109 (48.9)	57 (52.3)	
Active lifestyle	529 (49.4)	382 (51.7)	89 (39.9)	58 (53.2)	0.006*
Smoking habit					
Nonsmoker	1038 (96.9)	719 (97.3)	216 (96.6)	103 (94.5)	0.287
Former smoker	11 (1.0)	7 (0.9)	2 (0.9)	2 (1.8)	
Current smoker	22 (2.1)	13 (1.8)	5 (2.2)	4 (3.7)	
Family income					
Not enough	100 (9.3)	59 (8.0)	29 (13.0)	12 (11.0)	0.087
Enough but no saving	486 (45.4)	330 (44.7)	101 (45.3)	55 (50.5)	
Enough and saving	485 (45.3)	350 (47.4)	93 (41.7)	42 (38.5)	
History of chronic illness	126 (11.8)	90 (12.2)	17 (7.6)	19 (17.4)	0.028*
History of COVID-19 infection	(28.1) )301)	197 (26.7)	68 (30.5)	36 (33)	0.259
COVID-19 among acquaintances	766 (71.5)	533 (72.1)	164 (73.5)	69 (63.3)	0.123
COVID-19 related hospitalization among acquaintances	486 (45.4)	346 (46.8)	104 (46.6)	36 (33)	0.024*
COVID-19 related death among acquaintances	345 (32.2)	241 (32.6)	79 (35.4)	25 (22.9)	0.067

VA: vaccine acceptant; VH: vaccine hesitant; VR: vaccine resistant

^*^p value < 0.05

They were divided into 3 main groups according to their intention to get the COVID-19 vaccine into 3 groups: VA, VH and VR. Acceptability rate of COVID-19 vaccine was 69.0% of the study population, while VH group constituted 20.8% of the participants. However, 10.2% refused the vaccination.

**Fig. 2:**
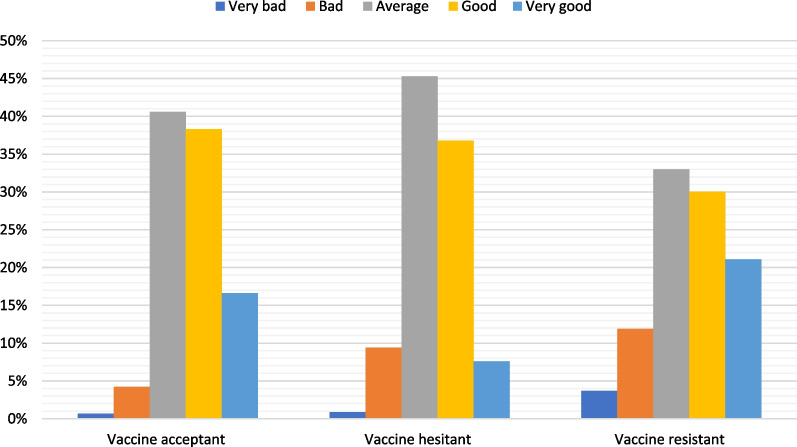
Self-rated knowledge level about COVID-19 among university students (*n* = 1071)

Figure 2 shows COVID-19 self-rated knowledge among the study groups. The responders rated their COVID-19 knowledge as average in 40.6%, 45.3% and 33.0% of VA, VH and VR groups, respectively.

On evaluating knowledge regarding COVID-19 vaccines (Table 2), participants showed a median knowledge score of 4 out of 8 (IQR = 8). Only 437 students (40.8%) answered correctly that the vaccine can be given to elderly. While around one-third of students (33.9%, *n* = 363) thought that the COVID-19 vaccine would not provide protection from the disease, almost half of the participants thought it would prevent the spread of the disease (49.6%, *n* = 531).

**Table 2:** Knowledge and beliefs of the university students about COVID-19 vaccination (*n* = 1071)

Variables Median (IQR) or n (%)	Total (*n* = 1071)	VA group (*n* = 739)	VH group (*n* = 223)	VR group (*n* = 109)	*p*
Knowledge score	4 (8)	4 (8)	4 (8)	3 (7)	< 0.001*
COVID‐19 vaccine is important	889 (83.0)	710 (96.1)	149(66.8)	30 (27.5)	< 0.001*
COVID‐19 vaccination to everyone in the community is important	812 (75.8)	682 (92.3)	110 (49.3)	20 (18.3)	< 0.001*
COVID‐19 vaccination should always be compulsory	589 (55.0)	518 (70.1)	61 (27.4)	10 (9.2)	< 0.001*
Concerns about COVID‐19 vaccination	750 (70.0)	439 (59.4)	214 (96.0)	97 (89.0)	< 0.001*
COVID‐19 vaccination of should always be compulsory for HCWs	833 (77.8)	661 (89.4)	141 (63.2)	31 (28.4)	< 0.001*
Approval of the vaccine guarantees its safety	829 (77.4)	414 (56.0)	50 (22.4)	7 (6.4)	< 0.001*
Vaccination is the best preventive measure for COVID‐19	665 (62.1)	578 (78.2)	78 (35.0)	9 (8.3)	< 0.001*
COVID‐19 vaccine may have adverse effects	829 (77.4)	514 (69.6)	212 (95.1)	103 (94.5)	< 0.001*
COVID‐19 vaccine may be ineffective	794 (74.1)	486 (65.8)	205 (91.5)	103 (94.5)	0.011*
You may get COVID-19 infection from the vaccine	501(46.8)	284 (38.4)	144 (64.6)	73 (67.0)	< 0.001*
If you get COVID-19 infection, you’re not at a risk of complications	638 (59.6)	459 (62.1)	118 (52.9)	61 (56.0)	0.036*
You are not at high risk to get Covid‐19 infection	520 (48.6)	339 (45.9)	118 (52.9)	63 (57.8)	0.023*

VA: vaccine acceptant; VH: vaccine hesitant; VR: vaccine resistant, IQR: interquartile range, HCW: health care workers

^*^P value < 0.05

**Fig. 3:**
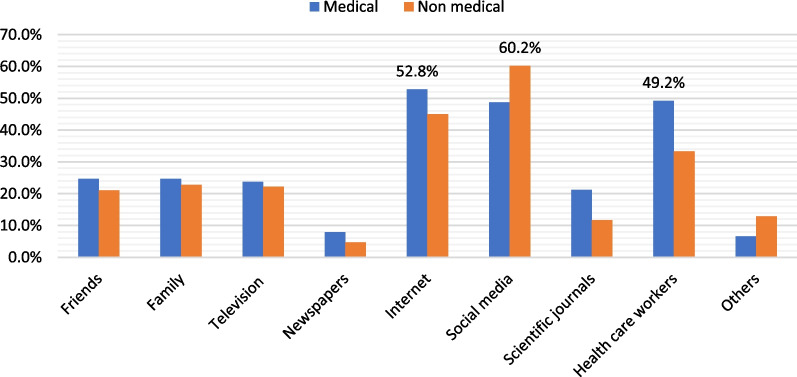
Sources of information about COVID-19 vaccine among medical and non-medical university students

The question about number of COVID-19 vaccine doses was answered correctly by 81.4% (*n* = 872) of the students. Although 44.4% of the participants (*n* = 476) knew that those previously infected with COVID-19 also need to take the vaccine, 33.5% (*n* = 359) chose to answer, “don’t know”. Also, only 487 participants (45.5%) answered correctly that COVID-19 vaccine should not be administered during COVID-19 infection. Lastly, more than half of the students (58.8%) reported that they don’t know if these vaccines can be given to children. Comparison of the study groups showed significant difference regarding knowledge scores between the three groups with the highest score in VA group. The students’ beliefs towards COVID‐19 vaccination are illustrated in Table 2. The main sources of information about COVID-19 vaccination among university students are shown in Fig. 3.

**Fig. 4:**
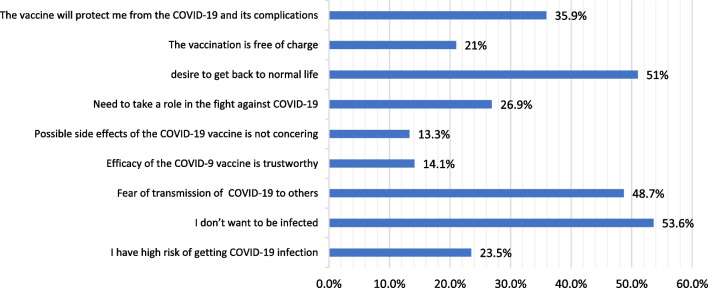
The perceived motivators for COVID‐19 vaccination among vaccine acceptant group (*n* = 739)

**Fig. 5:**
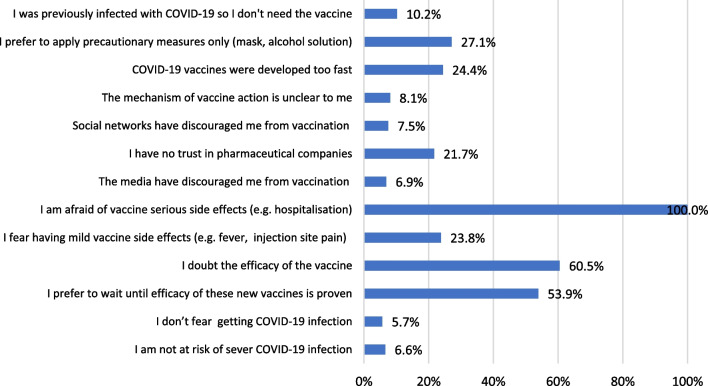
University students’ barriers explaining COVID-19 vaccine hesitancy or resistancy (*n* = 332)

**Fig. 6:**
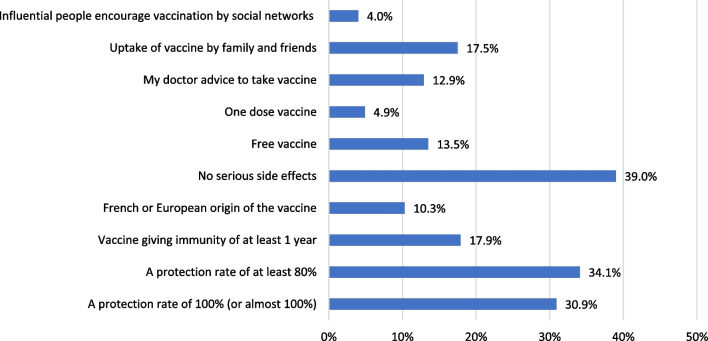
Reasons for change towards intention to get COVID-19 vaccination among vaccine hesitant group (*n* = 223)

Regarding the reasons for an intention to get COVID‐19 vaccination, 739 VA students reported that their main motives were fear of being infected (53.6%), desire to get back to normal life (51.0%) and fear of transmitting infection to others (48.7%) (Fig. 4). On the other hand, 332 students (VH and VR) declared that the main barriers against getting vaccinated were being afraid of serious side effects (100%) and doubting the efficacy of these vaccines (60.5%) as illustrated in Fig. 5. While VR refused to change their decision, VH group (*n* = 223) would change their decision if there is a low risk of serious side effects, or the protection rates reach 80–100% (Fig. 6).

As shown in Table 3, univariate and multivariate regression analysis revealed an increasing likelihood of vaccine acceptancy associated with an active lifestyle, a high knowledge score, and positive vaccine beliefs.

**Table 3:** Factors associated with the acceptance of a COVID-19 vaccine (*n* = 1071)

Variable	Univariate model		Multivariate model	
	Crude OR (95% CI)	*p*	Adjusted OR (95% CI)	*p*
Sex (female)	0.84 (0.63–1.11)	0.22	1.19 (0.80–1.78)	0.389
College (medical)	1.45 (1.03–2.04)	0.031*	1.20 (0.74–1.92)	0.463
Active lifestyle	1.35 (1.04–1.75)	0.025*	1.54 (1.07–2.22)	0.021*
Chronic illness	1.14 (0.76–1.71)	0.531	1.59 (0.91–2.78)	0.101
Knowledge score	1.53 (1.42–1.66)	< 0.001*	1.31 (1.18–1.46)	< 0.001*
COVID‐19 vaccine is important	20.93 (13.62–32.15)	< 0.001*	1.66 (0.88–3.11)	0.117
COVID‐19 vaccination to everyone in the community is important	18.59 )13.12–26.35)	< 0.001*	4.13 (2.41–7.08)	< 0.001*
COVID‐19 vaccination should always be compulsory	8.62 (6.35–11.70)	< 0.001*	1.79 (1.16–2.76)	0.008
Concerns about COVID‐19 vaccination	0.10 (0.06–0.16)	< 0.001*	0.21 (0.12–0.36)	< 0.001*
COVID‐19 vaccination of should always be compulsory for HCWs	7.88 (5.73–10.84)	< 0.001*	1.80 (1.15–2.82)	0.010
Vaccination is the best preventive measure for COVID‐19	0.71 (0.55–0.92)	0.009*	2.87 (1.95–4.23)	< 0.001*
If you get COVID-19 infection, you’re not at a risk of complications	10.11 (7.49–13.66)	< 0.001*	1.10 (0.77–1.58)	0.609
Constant	–	–	0.113	< 0.001

^*^p < 0.05

HCWs: health care workers

The last part of the questionnaire was about those who had already taken the COVID-19 vaccine (Table 4). Nearly one-fifth of students (21.0%) already received the COVID-19 vaccine. AstraZeneca and Sinopharm vaccines were the most administered vaccines (68.4%, 12.9%, respectively). Side effects after vaccination were reported by 146/225 students (64.9%) mostly in the form of widespread muscle/joint pain, headache, and fever.

**Table 4:** Types of the received COVID-19 vaccine and the experienced adverse effects in the university students vaccinated group (*n* = 225)

Variables	Vaccinated (*n* = 225) *n* (%)
Type of the received COVID-19 vaccine	
Pfizer-BioNTech	10 (4.4)
Moderna	3 (1.3)
AstraZeneca	154 (68.4)
Sinovac	21 (9.3)
Sinopharm	29 (12.9)
Sputnik V	3 (1.3)
Others	5 (2.2)
Experienced adverse effects of COVID-19 vaccine	146 (64.9)
Anaphylaxis	5 (2.2)
Skin rash	5 (2.2)
muscle/joint pain	143 (63.6)
Fever/chills	135 (60.0)
Fatigue/sleepiness	63 (28)
Chest pain/palpitations	31 (13.8)
Headache	118 (52.4)
Nausea	46 (20.4)
Vomiting	9 (4.0)
Poor appetite	40 (17.8)

## Discussion

It is of great importance to assess COVID-19 vaccine acceptance rates. This knowledge is essential for determining intervention measures and initiating communication campaigns. This would help in increasing the awareness and assurance of the public about vaccine safety and efficacy and result in limiting the spread of severe acute respiratory syndrome coronavirus 2 (SARS-COV2) [19, 20]. This study aimed at estimating the level of COVID-19 vaccine acceptance among university students in Egypt, their knowledge, and beliefs towards COVID-19 vaccination together with actual uptake of the vaccine. University students represent a unique subpopulation with various factors and habits, such as different thinking and paying more attention to media [11, 12]. Moreover, reports found increased rates of COVID-19 infection and subsequent hospitalization among young adults as their presence in academic and social settings makes them at a higher risk of being exposed to infection by SARS-CoV-2 [21]. Thus, measuring their acceptance towards receiving COVID-19 vaccine is essential for public health strategies.

The success of any vaccination program is related to both acceptance and uptake of this vaccine in the population. To achieve herd immunity and hinder the transmission of SARS-CoV-2. About 55.0% to 82.0% of the population should be vaccinated to stop transmission [22, 23].

Vaccination hesitancy is one of the primary problems that endangers public health especially during this COVID-19 pandemic that is associated with high mortality rates [24].

In this study, acceptability rate of COVID-19 vaccine was 69.0% of the university students while vaccine hesitancy was 20.8%. Fear of serious side effects and doubts about vaccine efficacy were the main barriers explaining hesitancy to uptake of COVID-19 vaccine. Poor level of knowledge was detected between university students towards COVID-19 vaccine. Actual uptake of vaccine was reported by 21.0% of the study university students.

Overall, 69.0% of participants were classified as VA, 20.8% were VH and 10.2% were classified as VR. These results are similar to those reported by Reiter et al. [6] where 69.0% of adult population in the US were VA and 31.0% were VR.. In Barello et al. [11], a study on university students from Italy, 86.1% were VA. In another study from France, 35.0% of the adults were qualified as VH [25]. In a cross-sectional study including 467 university students in the United Arab Emirates, an analytical approach was used. The acceptance rate of the COVID-19 vaccine among study participants was 56.3% [26]. In another Jordanian survey, 30.4% of the 2,208 participants stated that they would take a COVID-19 vaccine if it were available [27]. These differences are thought to be due to variations in geographical distribution, population characteristic, data collection, different periods of studies conduction, cultural disparities.

Vaccine hesitancy in females was much higher than males in our cohort. Female vaccine hesitancy is linked to a lower perceived risk of COVID-19, stronger trust in conspiracy theories regarding the pandemic compared to their male counterparts [28], and concerns about immunization safety during pregnancy and lactation [29].

Not surprisingly, most VA students (*n* = 633, 85.7%) were medical students. Several research were done to evaluate the acceptance of COVID-19 vaccines among medical students. A study reported 77.0% of medical students were VA [30] which is close to our results. A study on Egyptian medical students reported acceptability of 35.0% but that study was done on medical students from 2 university and was done at a different time point before start of vaccination in Egypt [17]. Manning et al. [31], on the other hand, reported lower incidence of VA in medical students (45.3%). In contrast, an Italian survey of students revealed no difference in vaccination acceptability between medical and non-medical students, which is highly concerning given that medical students should be better aware about health-related concerns than non-medical students [32].

About 75.8% and 77.8% of the students in this study thought that COVID-19 vaccine should be mandatory to the public and to healthcare workers (HCW), respectively. Lucia et al. [30] found similar results of 67.9%, 85.9%, respectively.

Around half (48.6%) of students thought they are not at elevated risk of acquiring COVID-19. Studies on nursing students [31, 33] reported that 26.2% and 31.8% perceived their risk to get infected with COVID-19 as extremely low or low, respectively. The students' high degree of vaccine hesitancy was interestingly linked to a similar high level of perception of an increased danger of contracting COVID19 infection. This conclusion was consistent with Lucia et al., 2020 [30] who found that more than 2 out of 10 students were vaccine hesitant despite a self-perception of an elevated risk of infection with COVID19. At the same time, this conclusion contradicts earlier research that found risk perception to be an important predictor of preventative intentions and protective health behaviors [34].

Almost half of the students (46.8%) were wrongly concerned they may get infected by COVID‐19 from the vaccine. Fewer percentage was (7.1%) reported by another study [31] which highlights presence of poor knowledge and misconceptions about the vaccine among university students, a problem which needs to be urgently addressed.

Also, 59.6% of respondents thought they would not be at risk of developing complications due to COVID-19 infection which is also reported by 52.5% of students in a previous study [31]. Comparison of the three group of this study regarding their belief of not developing complication on COVID-19 infection, the difference was statistically significant (*p* value = 0.03) which was found also in Manning et al. [31] study (*p* value = 0.02).

Poor knowledge about COVID-19 and its vaccines, found in our study, may contribute largely to vaccine hesitancy which has also been shown in some studies [31, 35, 36]. This poor knowledge indicates the necessity to provide university students with evidence-based education on the COVID-19 and its vaccines through webinars or courses and the vaccination program should include targeted educational campaigns.

In this study, medical students in this study mainly get information about COVID-19 vaccine from internet and health care workers. Another study reported that medical students [37] chose social media and public internet sites as their main source of information about COVID-19 vaccine. Information from unreliable sources would affect students’ decision towards vaccination. Health information collected from a variety of sources, including social media, may be fueling vaccine hesitancy. Individuals can quickly generate and share material around the world via social media without the need for editorial oversight. Furthermore, by utilizing vivid narratives and powerful imagery, social media may spread disinformation. Social media is also characterized by its ability to reach big audiences and disseminate information quickly [38].

The most frequently reported motivators for getting COVID-19 vaccination were desire to protect self, avoiding transmission to others, wanting to get back to normal life and the belief that it would be the best way for protection against COVID-19 and its complication. This was in agreement with Manning et al. study [31].

Fear and suspicion in addition to lack of information about vaccine development led to COVID-19 vaccines hesitancy [39]. In the present study, (70.0%) of all participants had concerns about the COVID-19 vaccine especially in the VH group as 96.0% of them had fears about receiving it. A study by Hatmal et al. [40] showed that 53.0% of all the participants were scared to have the vaccine. The main reasons behind vaccine hesitancy and refusal in this study were fear of side effects, doubting the efficacy and safety of vaccine, preferring to wait until further studies as the development of vaccine was fast. These concerns were reported by other studies as well [31, 33, 41–43].

In VH group, 95.1% believed that COVID-19 vaccine may have side effects, 91.5% thought it would be ineffective. A study reported similar rates of hesitancy as 89.2% were worried about side effects 83.8% thought it would be ineffective [30].

A large study [44] conducted on 32,361 adults are in agreement with our study as they suggested that the main factors against receiving a COVID-19 vaccine were mistrust in the safety of vaccines and concerns about their effects.

As of 15^th^ of September 2021, more than 5 billion doses of COVID-19 vaccine have been administered worldwide. As for the situation in Egypt, on 16 September 2021, there have been nearly 294,000 cases of COVID-19 infection with 17,000 deaths and a total of 12 million administered vaccine doses. About Four and half million persons fully vaccinated and almost 8 million persons received at least one dose. Thus, nearly 8.0% of the population is being vaccinated [5]. As of 17 December 2022, a total of 100,993,230 vaccine doses have been administered in Egypt [45].

Vaccinated participants received mainly AstraZeneca, Sinopharm and Sinovac vaccines which is logical since they were the initial vaccines approved in Egypt at beginning of vaccination with other types being sequentially added to the list of approved vaccines [5].

Side effects after vaccine were reported by 146 out of 225 vaccinated students (64.9%) mostly in the form of widespread muscle/joint pain, headache and fever as in line with other studies [40, 46, 47].

This study has many strengths. First, the large sample size, recruiting participants from all over Egyptian universities. Second, examination of a lot of variables and the timeframe of the study was after beginning of administration of vaccines in Egypt but before it became mandatory to university students which is considered a good point for this research as we measured vaccine acceptance while the vaccines were voluntarily taken. This information would be of great value to identify the gaps and implement appropriate training and interventions.

The main limitation of this study is that students were still down on the priority list for vaccination after elderly and HCW. Thus, only small part was vaccinated. But, after our study was finished, vaccination became mandatory to university students. Also, this is a cross-sectional survey study without follow up of participants to monitor variations in vaccine intention and adverse effects after vaccination. Moreover, as participation in this study was voluntary, gender balance could not be achieved. lastly, the study was done using a web-based survey distributed through social media where participants needed access to a smartphone, tablet, or computer to participate, hence, the study may miss people from lower socioeconomic classes which may cause a selection bias. However, this was the only appropriate method to collect data from the university students as the study was in the time of summer vacation and to avoid direct contact with them to avoid the spread of COVID-19, specially that the study period coincided with the surge of 4^th^ wave of COVID-19 pandemic in Egypt.

## Conclusions

This study highlights the high acceptance rates of COVID-19 vaccination among university students in Egypt. Poor knowledge levels were detected regarding COVID-19 vaccines. Fear of side effects, doubting the effectiveness and safety of vaccine were the main reasons behind vaccine hesitancy. Vaccine acceptability is associated with an active lifestyle, a high knowledge score and positive vaccine beliefs.

We recommend monitoring trends in vaccine acceptability and determine how that reflects into vaccine uptake rates. Healthcare providers can play a key role in promoting for the uptake of a COVID-19 vaccine. Students represent a good target for educational campaigns and efforts should be made to improve medical education of university students by implementing vaccinations and preventive behaviors courses for all university students specially to medical students. Implementation of vaccination policies, raising campaigns to increase knowledge about vaccine benefits are highly recommended.

## Data Availability

The datasets generated during and/or analyzed during the current study are available from the corresponding author on reasonable request.

## Abbreviations

CDC: Center of Disease prevention and Control
COVID-19: Coronavirus disease 2019
SARS-CoV-2: Severe acute respiratory syndrome coronavirus 2
VA: Vaccine acceptant
VH: Vaccine hesitant
VR: Vaccine resistant
WHO: World Health Organization
